# Tongxinluo Regulates Expression of Tight Junction Proteins and Alleviates Endothelial Cell Monolayer Hyperpermeability via ERK-1/2 Signaling Pathway in Oxidized Low-Density Lipoprotein-Induced Human Umbilical Vein Endothelial Cells

**DOI:** 10.1155/2017/4198486

**Published:** 2017-03-19

**Authors:** Chengcheng Chang, Hongli Liu, Cong Wei, Liping Chang, Junqing Liang, Hongying Bei, Hongrong Li, Shen Liu, Yiling Wu

**Affiliations:** ^1^College of Basic Medicine, Nanjing University of Chinese Medicine, Nanjing 210023, China; ^2^State Key Laboratory of Collateral Disease Research and Innovation Medicine, Shijiazhuang 050035, China; ^3^2nd City Hospital, Shijiazhuang 050051, China; ^4^Key Laboratory of State Administration of TCM (Cardio-Cerebral Vessel Collateral Disease), Shijiazhuang 050035, China; ^5^Department of Pharmacology, Hebei Yiling Pharmaceutical Research Institute, Shijiazhuang 050035, China; ^6^Graduate School, Hebei Medical University, Shijiazhuang 050017, China; ^7^Dongzhimen Hospital, Beijing University of Chinese Medicine, Beijing 100700, China; ^8^Key Disciplines of State Administration of TCM for Collateral Disease, Shijiazhuang 050035, China

## Abstract

Vascular hyperpermeability resulting from distortion of endothelial junctions is associated with a number of cardiovascular diseases. Endothelial tight junction regulates the paracellular permeability of macromolecules, a function of* Human Umbilical Vein Endothelial Cells* (HUVEC) monolayers that can be regulated by* oxidized Low-density Lipoprotein* (ox-LDL). However, the understanding of drug regulation of vascular hyperpermeability is so far limited. This study thus aimed to investigate the role of* Tongxinluo* (TXL) in the maintenance of the vascular endothelial paracellular permeability. Here, changes in permeability were determined by measuring the paracellular flux of FITC-dextran 40000 (FD40), while protein expression and intercellular distribution were examined by western blot and immunofluorescence assay, respectively. We found that TXL alleviated the ox-LDL-induced increase in flux of FD40 and then reduced the hyperpermeability. Moreover, ox-LDL-induced disruptions of ZO-1, occludin, and claudin1 were also restored. This is via the activation of ERK1/2 in the vascular endothelial cells. Our results provide insights into the molecular mechanism by which TXL alleviates ox-LDL-induced hyperpermeability and provide the basis for further investigations of TXL as regulators of vascular barrier function.

## 1. Introduction 

The vascular endothelial barrier function plays a critical role in the maintenance of the homeostasis in the body. Endothelial cells covering the luminal surface of blood vessels serve as an interface between circulating blood and surrounding tissues. Tight junctions provide structural integrity to tissues and create highly polarized barriers with selective paracellular permeability to water, solutes, and larger molecules. Tight junctions are comprised of different types of transmembrane proteins including occludin and claudins and membrane-associated proteins, such as ZO-1, which link the junctional membrane to the cytoskeleton to regulate endothelial barrier function [1]. Cumulative reports indicate that the lack or redistribution of any of tight junction associated proteins compromises the barrier function. It has been shown that ZO-1, as well as indeed other submembrane proteins (ZO-2, ZO-3, etc.), interacts directly with occludin and claudins and associates with the adherens junctions as well as gap junctions [2]. Thus, ZO-1 appears to be crucial in the regulation of the formation and function of endothelial barrier. Recent studies have shown that ZO-1 and JAM-A form a cooperative unit that stimulates junctional actomyosin activation and thereby regulates endothelial barrier formation [3]. Occludin and claudins, the key components of the transmembrane proteins in the tight junction protein complex, have been implicated in the regulation of barrier function [4]. Although the formation of tight junction does not necessarily require occludin [5], it seems to play a permeability regulating role by incorporating itself into the claudin-based strand [6]. Research has shown that downregulation of occludin via either proteolysis or treatment with permeabilizing factors, vascular endothelial growth factor (VEGF), or phospholipase D2 (PLD2) is directly linked to elevated endothelial permeability [7]. It has been confirmed that the main function of claudins is to generate tight junction strands, the structural and functional core of tight junctions within the plasma membrane. Besides, the redistribution or decrease expression of claudins, such as claudin1, is linked to hyperpermeability in endothelial cells [8, 9].

It has been recognized that* oxidized Low-density Lipoprotein-* (ox-LDL-) induced dysfunction of the vascular endothelium is one of the key factors in the pathogenesis of many cardiovascular diseases such as hypertension [10], atherosclerosis, and coronary heart disease [11, 12]. It is further established that ox-LDL is able to increase vascular permeability to serum proteins both in vivo [13, 14] and in vitro [15, 16], one of the functional behaviors of early endothelial dysfunctions induced by ox-LDL. Ox-LDL increases endothelial permeability by causing a cytoskeleton-dependent contraction of the endothelial cells and opening of intracellular gaps, which then allow large components such as proteins and monocytes to trespass the endothelial barrier [15, 17]. However,whereas endothelial tight junction is known as an important regulator in the paracellular permeability of endothelial cells, the relevant mechanism of HUVEC damaged by ox-LDL remains to be fully understood.

*Tongxinluo* (TXL), a traditional Chinese herbal medicine, was registered in the State Food and Drug Administration of China in 1996. TXL is composed of* Radix ginseng, Buthus martensi, *Hirudo*, Eupolyphaga seu steleophaga*,* Scolopendra subspinipes*,* Periostracum cicadae*,* Radix paeoniae rubra*,* Semen ziziphi spinosae*,* Lignum dalbergiae odoriferae*,* Lignum santali albi*, and* Borneolum syntheticum* and has been used for unstable angina pectoris and acute coronary syndrome treatment in China [18, 19]. Previous researches have indicated that TXL has multiple functions, including improvement of endothelial cell function, reduction of lipid levels, vasodilation, anti-inflammation, antiapoptosis, and enhancement of angiogenesis [20–22]. Recent studies have reported that TXL has influence on the disruption of tight junction proteins in endothelial cells and on improving endothelial barrier function [23–25]. Several studies have implicated that the ERK1/2 pathway could be involved in the modulation of paracellular permeability through altering the expression of several tight junction proteins [26]. It was reported that activation of ERK1/2 was involved in tight junction disruption of HUVECs induced by hydrogen peroxide (H_2_O_2_), tumor necrosis factor-*α* (TNF-*α*) [27, 28]. Hence, we hypothesized that TXL may be involved in alleviating the paracellular permeability of endothelial cells, by suppressing the destruction of vascular endothelial barrier function. In this study, ox-LDL was used to induce the hyperpermeability of HUVEC monolayers, and TXL was found to alleviate such hyperpermeability via the ERK1/2 signaling pathway.

## 2. Material and Methods

### 2.1. Materials

*TXL Preparation*. TXL, the superfine powder, was purchased from Shijiazhuang YiLing Pharmaceutical Company (Shijiazhuang, China). The herbal drugs were authenticated and standardized on marker compounds according to the Chinese Pharmacopoeia, 2005. TXL was weighed and dissolved in the serum-free DMEM medium. Aided by ultrasound technology, the medicine was dissolved in about 0.5 h. The drug was then centrifuged at 10000 ×g for 10 min. The supernatant was filtered (0.22 *μ*m pore size) and then calculated for the final concentration, before being stored at −20°C for future use.

Antibodies against occludin and claudin1 were purchased from Abcam (Cambridge, UK). Antibodies against ZO-1, ERK1/2, and phosphorylated ERK1/2 were purchased from Cell Signaling Technology (CST) (Danvers, USA). PD98059 was purchased from Selleck (Houston, USA). Transwell permeable supports were purchased from Corning (New York, USA). FITC-dextran 40000 (FD40) was purchased from Sigma (Dorset, UK).

### 2.2. Culture of HUVECs

Primary HUVECs were purchased from Cell Bank of the Chinese Academy of Sciences (Shanghai, China). The cells were collected and cultured in DMEM that contained 10% fetal bovine serum, 2 mM glutamine, and 1% penicillin/streptomycin in an incubator at 37°C with 5% CO_2_.

### 2.3. Cell and Western Blot Analysis

HUVECs were coincubated with TXL for 4 h and then with PD98059 for 0.5 h. The cells were then exposed to ox-LDL for 24 h. Cell lysis was prepared in 1% sodium dodecyl sulfate (SDS) lysis buffer (1% SDS, 10 mM Tris/HCL [pH 7.2], and 1.0 mM Na_3_VO_4_). Cellular protein concentrations were quantified using BCA assay. Protein samples were boiled in SDS loading buffer, run on 10% or 12% SDS polyacrylamide gel, transferred to Nitrocellulose Blotting membrane (Life Sciences, Mexico), and blocked with Odyssey® blocking buffer (PBS) (LI-COR, Lincoln, USA) for 2 h at 25°C. Primary antibody incubations were performed overnight at 4°C. Secondary antibodies (1 : 5000) (Abcam, Cambridge, UK) were incubated for 1 h at 25°C. Then the membrane was scanned by Odyssey (LI-COR, Lincoln, USA).

### 2.4. Immunofluorescence Assay

HUVECs were seeded onto flame-sterilized coverslips that were placed in a 24-well tissue culture plate. The cells were treated with or without PD98059 for 0.5 h after being incubated with or without TXL for 4 h, and then 120 *μ*g/mL ox-LDL was added to the wells for 24 h. The cells were fixed for 10 min in methyl alcohol or 4% (w/v) paraformaldehyde and then made permeable with 0.25% Triton X100 for 5 min. Blocking solution was added at 25°C for 1 h. Then anticlaudin1 (1 : 200), antioccludin (1 : 200), and anti-ZO-1 (1 : 200) antibodies were added to each well overnight at 4°C, and the secondary antibodies (1 : 200) were added and incubated for 1 h at 25°C. The cells were then incubated with DAPI for 5 min to stain the nucleus. The cells were imaged using a ZEISS confocal microscopy (Oberkochen, Germany).

### 2.5. In Vitro Vascular Permeability Assay

HUVECs were seeded on the upper chamber of the polycarbonate membrane transwell inserts with 0.4 *μ*m pores and allowed to grow to full confluence for the formation of a monolayer. The upper chamber was filled with 200 *μ*L of media, and the lower chamber was filled with 850 *μ*L of media. HUVECs were pretreated with TXL (200 *μ*g/mL) for 4 h before being stimulated with ox-LDL (120 *μ*g/mL) for 24 h. Then, 5 *μ*L of FD40 (2 mg/mL) was added to basal media and allowed to permeate through the monolayers, and 100 *μ*L samples were taken from lower chamber at 0 h, 1 h, 2 h, 4 h, 8 h, 12 h, and 24 h, respectively. The collected samples were transferred to 96-well black plate. Fluorescence intensities for each group were measured using a fluorescence microplate reader (BioTek, Winooski, USA) with 485 nm excitation and 535 nm emission wavelength and compared with control group.

### 2.6. Statistical Analysis

All results are presented as means ± SD from three or more independent experiments. Statistical significance was assessed by one-way ANOVA for comparison of different groups. *P* <  0.05 and *P* < 0.01 represent statistically significant differences.

## 3. Result

### 3.1. TXL Inhibited Ox-LDL-Induced Increase in Permeability of the HUVEC Monolayer

**Figure 1:**
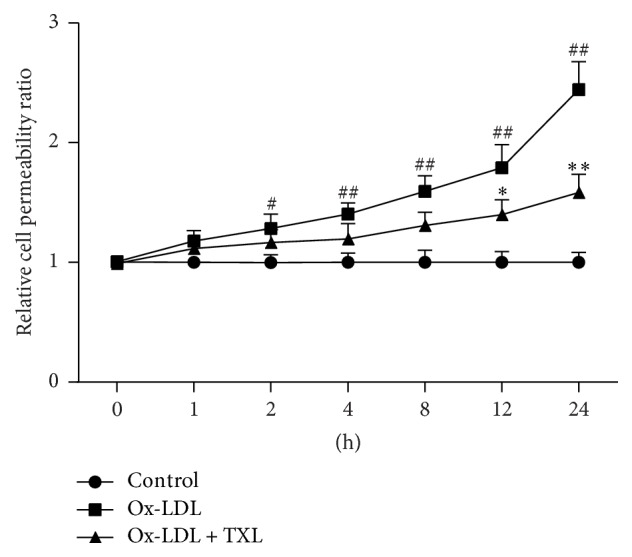
TXL decreased ox-LDL-induced hyperpermeability in HUVECs. HUVECs were treated with ox-LDL (120 *μ*g/mL) for 24 h prior to treatment with TXL (200 *μ*g/mL) for 4 h. At different time points, the endothelial permeability was determined by the transwell assay. Quantitative analysis was performed by measuring FD40 fluorescence intensity relative to the control. The data were obtained from three independent experiments. Each bar represents the mean (±SD) of three independent experiments. ^#^*P* < 0.05, ^##^*P* < 0.01 versus control, ^*∗*^*P* < 0.05, and ^*∗∗*^*P* < 0.01 versus ox-LDL group.

HUVECs were treated with ox-LDL (120 *μ*g/mL) for 24 h, and endothelial permeability was measured by FD40 fluorescence intensity from the lower chamber. Transwell assay data revealed that ox-LDL significantly raised endothelial permeability in a time-dependent manner (Figure 1).

However, when HUVECs were pretreated with TXL for 4 h and then challenged by ox-LDL for another 24 h, it was very interesting to note that TXL decreased hyperpermeability induced by ox-LDL. This reduction was highly significant in 12 and 24 hours after the treatment. According to the above results, we determined HUVECs exposure to ox-LDL for 24 h in the experiment shown in Figure 1.

### 3.2. TXL Improved Tight Junction Proteins Expression in Ox-LDL-Induced HUVEC Cells

**Figure 2:**
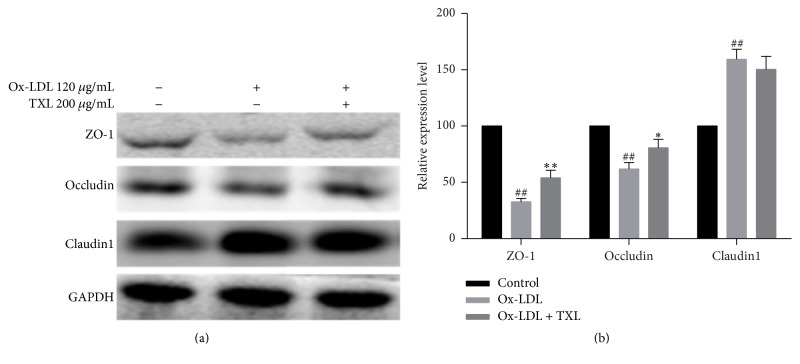
TXL improved ox-LDL-induced tight junction proteins expression in ox-LDL-induced HUVEC cells. HUVECs were treated with ox-LDL (120 *μ*g/mL) for 24 h prior to treatment with TXL (200 *μ*g/mL) for 4 h. Western blot analysis of ZO-1, occludin, claudin1, and GAPDH was performed (a). GAPDH served as the loading control. Quantitative analysis was performed by measuring protein expression relative to the control. The data were obtained from three independent experiments (b). Each bar represents the mean (±SD) of three independent experiments. ^##^*P* < 0.01 versus control, ^*∗*^*P* < 0.05, and ^*∗∗*^*P* < 0.01 versus ox-LDL group.

To further explore the action underlying TXT effect on paracellular permeability, we evaluated the effect of TXL on tight junction proteins expression in ox-LDL-treated HUVECs with Western blot analysis. Ox-LDL exposure for 24 h caused a significant decrease in the protein levels of occludin and ZO-1. However, the expression of claudin1 was markedly increased. When HUVECs were pretreated with TXL before ox-LDL exposure for 24 h, the decrease in occludin and ZO-1 expression in ox-LDL stimulated HUVECs was significantly augmented by TXL. It is interesting to note that TXL had no distinct effect on claudin1 expression compared with ox-LDL group (Figure 2).

### 3.3. Involvement of ERK1/2 Signaling Pathway in TXL Prevented Ox-LDL-Induced Hyperpermeability

To determine the regulatory mechanisms of TXL involved in tight junction proteins expression in HUVECs, the ERK1/2 inhibitor PD98059 was added to cultures 0.5 h before the TXL and ox-LDL treatments.

#### 3.3.1. Western Blot Analysis

**Figure 3:**
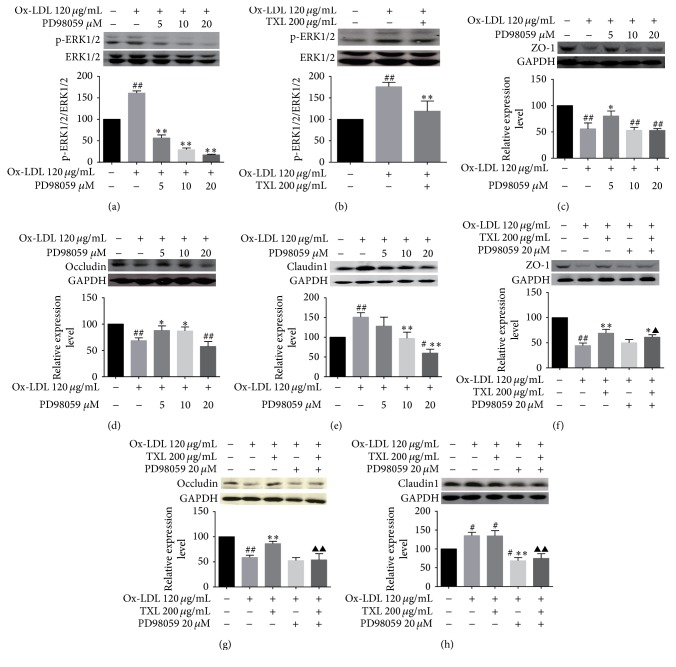
TXL or PD98059 inhibited ox-LDL-induced ERK1/2 signaling pathway and the effect on tight junction proteins expression. HUVECs were treated with ox-LDL (120 *μ*g/mL) for 24 h, prior to treatment with TXL (200 *μ*g/mL) for another 4 h, with or without incubation with PD98059 (20 *μ*M) for 0.5 h. Western blot analysis for ERK1/2, p-ERK1/2, ZO-1, occludin, claudin1, and GAPDH was performed. GAPDH served as the loading control. Quantitative analysis was performed by measuring protein expression relative to the control. The data were obtained from three independent experiments. Each bar represents the mean (±SD) of three independent experiments. ^*∗*^*P* < 0.05, ^*∗∗*^*P* < 0.01 versus ox-LDL group, ^#^*P* < 0.05, and ^##^*P* < 0.01 versus control. ^▲^*P* < 0.05, ^▲▲^*P* < 0.01 versus ox-LDL + TXL group.

We first investigated the influence of PD98059 and TXL on the phosphorylation levels of ERK1/2 which were activated by ox-LDL. Western blot analysis demonstrated that the phosphorylation levels of ERK1/2 were significantly increased in the ox-LDL-treated group but clearly abrogated by PD98059 in a dose-dependent manner (Figure 3(a)). TXL treatment partly prevented ox-LDL-induced activation of ERK1/2 (Figure 3(b)).

To investigate the effect of different phosphorylation levels of ERK1/2 on tight junction proteins and based on the dose response curve, selected different doses of PD98059 in the subsequently tests, namely, low dose (5 *μ*M) and high dose (20 *μ*M), respectively, had marginal and marked inhibitory effect on ERK1/2 phosphorylation (Figure 3(a)). Results showed that PD98059 had a significant improvement on the expressions of ZO-1 (treatment with PD98059 5 *μ*M, Figure 3(c)) and occludin (treatment with PD98059 5 or 10 *μ*M, Figure 3(d)) in the ox-LDL-induced cell, whereas the effect was eliminated with the phosphorylation ERK 1/2, almost completely inhibited with PD98059 20 *μ*M (Figures 3(c), and 3(d)). Interestingly, the activation levels of ERK1/2 were inhibited by PD98059 in a dose-dependent manner; the expression of claudin1 was also decreased in the same way (Figure 3(e)).

To investigate the role of ERK1/2 signaling pathway in the influence of TXL on the tight junction protein expression, we inhibited ERK1/2 further with PD98059 20 *μ*M. PD98059 20 *μ*M significantly neutralized the effect of TXL on the expression of ZO-1, but it still had a positive effect compared with ox-LDL group (Figure 3(f)). In terms of occludin expression, the improvement of TXL was abolished completely compared with ox-LDL + TXL group, similar to the ox-LDL group (Figure 3(g)). With the PD98059 treatment, there was a significantly decreased level of caudin1 expression both in TXL and ox-LDL-induced HUVECs, which were seen below the levels in the control group (Figure 3(h)).

#### 3.3.2. Immunofluorescent Staining Assay

We also performed immunofluorescent staining for tight junction proteins to analyze their cellular distribution in response to ox-LDL, TXL, and PD98059.

**Figure 4:**
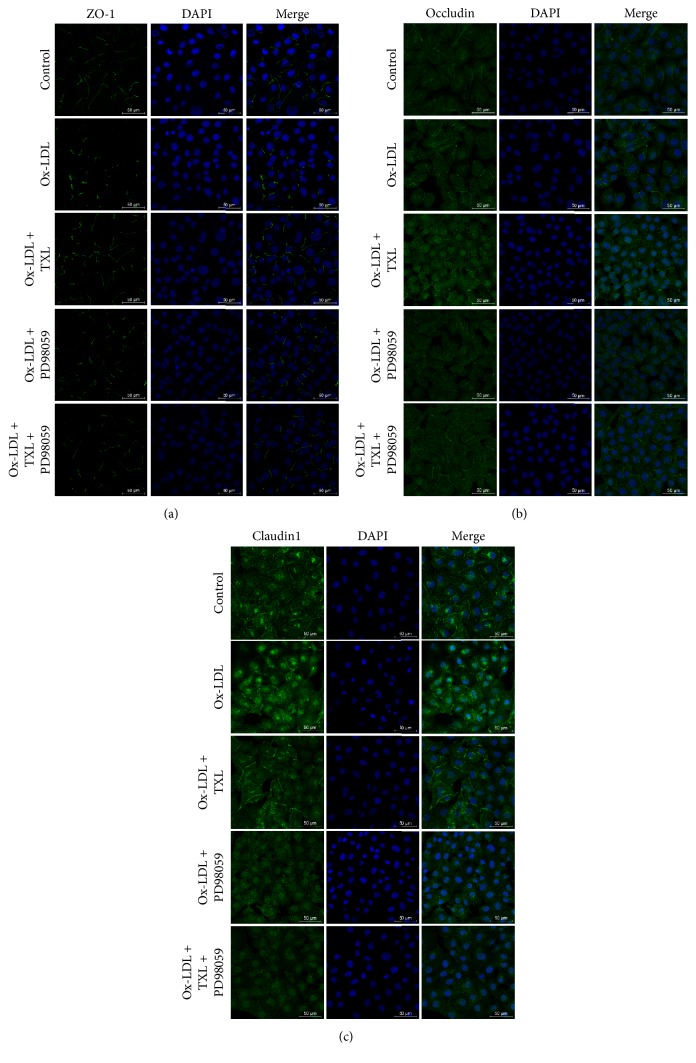
TXL restored ZO-1, occludin, and claudin1 expression in ox-LDL-treated HUVECs. HUVECs were treated with ox-LDL (120 *μ*g/mL) for 24 h, prior to treatment with TXL (200 *μ*g/mL) for another 4 h, with or without incubation with PD98059 (20 *μ*M) for 0.5 h. Immunofluorescent staining detection of ZO-1 (a), occludin (b), and claudin1 (c) expression.

Continuous distribution of occludin, ZO-1, and claudin1 was observed in the cell-cell junctions areas in control HUVEC cells. Treatment with ox-LDL disrupted the distribution of occludin, ZO-1, and claudin1, which appeared as discontinued spots between the cells. The staining of ZO-1 and occludin was also seen to reduce even disappear from regions of intercellular contact. In contrast, ox-LDL exposure rendered claudin1 staining visible in both cytoplasmic and nucleus regions (Figures 4(a), 4(b), and 4(c) ox-LDL groups). TXL treatment minimized ox-LDL-induced disruption of the tight junction structure, as seen by an increase in ZO-1 and occludin staining and enhancement of liner distribution within intercellular contact including claudin1 (Figures 4(a), 4(b), and 4(c) ox-LDL + TXL groups). Pretreatment with PD98059 in ox-LDL groups partly reversed the discontinuous distribution of ZO-1; it nonetheless minimized the effect of TXL (Figure 4(a) ox-LDL + PD98059 group, ox-LDL + TXL + PD98059 group). This observation was also made with occludin staining (Figure 4(b) ox-LDL + PD98059 group, ox-LDL + TXL + PD98059 group). It was noteworthy that PD98059 incubation resulted in claudin1 staining almost entirely disappeared both in ox-LDL + PD98059 group and ox-LDL + TXL + PD98059 group (Figure 4(c)).

#### 3.3.3. Transwell Assay Analysis

**Figure 5:**
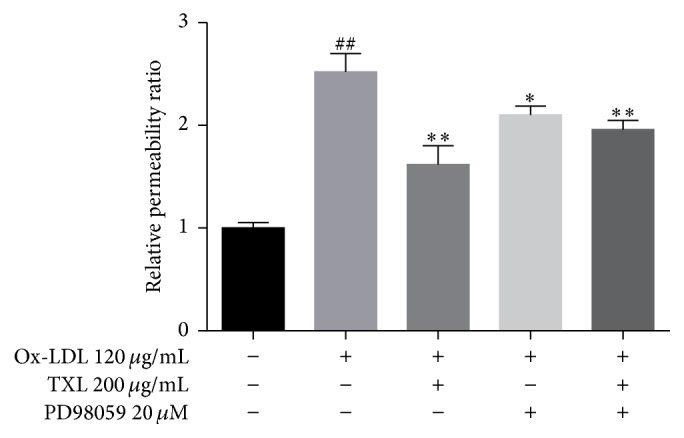
TXL decreased ox-LDL-induced hyperpermeability. HUVECs were treated with ox-LDL (120 *μ*g/mL) for 24 h, prior to treatment with TXL (200 *μ*g/mL) for another 4 h, with or without incubation with PD98059 (20 *μ*M) for 0.5 h. The endothelial permeability was determined by the transwell assay. Quantitative analysis was performed by measuring FD40 fluorescence intensity relative to the control. The data were obtained from three independent experiments. Each bar represents the mean (±SD) of three independent experiments. ^*∗*^*P* < 0.05, ^*∗∗*^*P* < 0.01 versus ox-LDL group, and ^##^*P* < 0.01 versus control.

Endothelial permeability was measured by FD40 fluorescence intensity from the lower chamber of transwell assay. HUVECs were treated with ox-LDL (120 *μ*g/mL) for 24 h, prior to treatment with TXL (200 *μ*g/mL) for another 4 h, with or without incubation with PD98059 (20 *μ*M) for 0.5 h. Transwell data showed that TXL significantly decreased ox-LDL-induced endothelial permeability after incubation for 24 h (Figure 5). PD98059 significantly reduced the permeability as a result of the ox-LDL exposure, but it also receded the effect of TXL.

## 4. Discussion

TXL consists of 12 herbs and insects and represents a traditional Chinese formula widely used to treat cardiovascular diseases in China. So far, TXL has been used to treat patients with angina pectoris for about two decades in clinical practice [29]. Biologically, TXL has pleiotropic effects, such as improvement of endothelial cell function, reduction of lipid levels, vasodilation, anti-inflammation, antiapoptosis, and enhancement of angiogenesis. All these effects could in fact be attributable to the endothelial barrier protection of TXL.

It has been reported that ox-LDL enhances monocyte transmigration of the endothelium and thus plays a critical role in the pathophysiological modulation of EC functions. Ox-LDL induces endothelial activation and injury resulting in generation of reactive oxygen species and an inflammatory response which leads to recruitment, activation, and migration of monocytes through interendothelial gaps to the subendothelial region [30, 31]. In addition, the transmigration of leukocytes in turn increases the opening of endothelial cells junctions [32]. Consequently, these effects may contribute to the high permeability in ox-LDL treated HUVECs. Our data also showed that ox-LDL indeed induces high permeability of FD40 in HUVECs.

Endothelial tight junction protein regulates the paracellular permeability of endothelial cells. In the present study, we investigated the effects of ox-LDL on the expression of ZO-1, occludin, and claudin1 in HUVEC and explored the protective effect of TXL on the endothelial function. We have provided evidence that ox-LDL appears to increase permeability in vitro, involving the activation of ERK1/2 signaling pathway. This is also connected with a subsequent disruption of ZO-1, occludin, and claudin1 from the endothelial cell-cell junctions. Here, we have firmly demonstrated that ox-LDL caused an aberrant distribution and a significant protein expression change of ZO-1, occludin, and claudin1 in cell-cell junction area. It is clear that these changes have contributed to the upregulation of the paracellular permeability in the endothelial cells [9, 33, 34]. However, the hyperpermeability was significantly diminished when the cells were pretreated with TXL. Our data clearly show that this effect of TXL is also linked to ZO-1 and occludin expression (but not claudin1).

To further investigate the role of ERK1/2 pathway in the ox-LDL-induced HUVECs treated with TXL, PD980598 was used as an inhibitor to the pathway in our study. It seems that the expression of ZO-1 and occludin is dependent on the degree of ERK1/2 phosphorylation. Our results indicate that ERK1/2 signaling pathway may have bidirectional action on the expression of ZO-1 and occludin. Namely, either overactivation or complete inhibition could downregulate the expression of ZO-1 and occludin. TXL has a significant inhibitory effect (does not completely inhibit) on the phosphorylation of ERK1/2 level compared with the ox-LDL group (western blot), and this might be the main reason of the expression of ZO-1 and occludin upregulation. At 20 *μ*M, PD980598 completely blocked the phosphorylation of ERK1/2 and had no influence on ZO-1 and occludin protein levels (western blot) in ox-LDL-induced HUVECs, although there was a slight improvement in ox-LDL-induced abnormal ZO-1 (IF). Again at 20 *μ*M, the effect of TXL on ZO-1 and occludin was significantly suppressed by PD98059, indicating that TXL played an effective role in ZO-1 and occludin expression which might rely on ERK1/2 signaling pathway.

One of the major limitations of the current study is the compound mixture of the TXL extracts, of which the contributing component(s) to the observed protective effects are not defined. This will be a future project and challenge. However, a possibility exists that the cumulative or synergistic effects of multiple compounds present in the herbal extract may jointly contribute to the effect and again this will be our next step to take in this study.

## 5. Conclusions

In conclusion, we found that ERK1/2 was principally responsible for the expression of tight junction proteins induced by ox-LDL. This study showed that activating ERK1/2 signaling using ox-LDL disrupted and decreased ZO-1 and occludin distribution and expression. Although activating ERK1/2 signaling using ox-LDL increased claudin1 expression, the distribution is disrupted. All these results are with subsequent increasing cell permeability. TXL partly normalized ox-LDL-induced disruption and distribution of tight junction proteins and prevented ox-LDL-induced hyperpermeability by modulating the activation of ERK1/2 pathway. Our findings also suggest that ERK1/2 may represent a new target molecule for protecting EC monolayer barrier functions in ox-LDL-induced HUVEC with TXL treatment. Taken together, TXL can improve ox-LDL-induced disruption of tight junctions and consequently lessens damage to the permeability of the HUVEC monolayer. However, more in vivo experimental studies are still in need to verify the effects of TXL.
